# Meteorological factors associated with hand, foot and mouth disease in a Central Highlands province in Viet Nam: an ecological study

**DOI:** 10.5365/wpsar.2017.8.1.003

**Published:** 2019-12-13

**Authors:** Hau Van Pham, Uyen Thi Ngoc Phan, Anh Nguyen Quynh Pham

**Affiliations:** a Hong Bang International University, Ho Chi Minh City, Viet Nam.; b Pasteur Institute in Ho Chi Minh City, Ho Chi Minh City, Viet Nam.; c University of Medicine and Pharmacy, Ho Chi Minh City, Viet Nam.

## Abstract

**Background:**

Hand, foot and mouth disease (HFMD) is a public health problem in Viet Nam, and studies have reported seasonal fluctuation in the occurrence of HFMD. This study sought to describe the occurrence of HFMD and its associated meteorological factors in Dak Lak province, Viet Nam.

**Methods:**

Monthly data on HFMD cases were collected from all commune health stations in Dak Lak province from 2012 through 2013. An HFMD case was defined as a brief febrile illness accompanied by a typical skin rash with or without mouth ulcers. Average temperature, maximum temperature, minimum temperature, humidity, rainfall, evaporation, sunshine duration and wind speed were recorded monthly at five local meteorological stations throughout Dak Lak.Data were aggregated at the district level, and the association between these meteorological factors and HFMD cases were examined by Poisson regression.

**Results:**

In 2012 through 2013, there were 7128 HFMD patients in Dak Lak. The number of HFMD cases increased during the rainy season. An increased risk of HFMD was associated with higher average temperature (risk ratio and 95% confidence interval: 1.06; 1.03–1.08 per 1 °C increase), higher rainfall (1.19; 1.14–1.24 per 200 mm increase) and longer sunshine duration (1.14; 1.07–1.22 per 60 hours increase). The risk of HFMD was inversely associated with wind speed (0.77; 0.73–0.81 per 1 m/s increase).

**Conclusion:**

This study suggests that there is a significant association between HFMD occurrence and climate. Temperature, rainfall, wind speed and sunshine duration could be used as meteorological predictors of HFMD occurrence in Viet Nam’s Central Highlands region. Intensified surveillance for HFMD during the rainy season is recommended.

Hand, foot and mouth disease (HFMD) is an acute enterovirus infectious disease. HFMD has no vaccine or specific therapy thus far. Early detection of outbreaks, early recognition of severe HMFD and timely supportive treatment are among the key principles applied to minimize the burden of disease. (*1*) HFMD is a major health problem in many countries, notably in the World Health Organization’s (WHO) Western Pacific Region, including Viet Nam. (*1*-*3*) In Viet Nam, the first HFMD epidemic was reported in Ho Chi Minh City in 2003, (*4*) it then gradually spread around the country until multiple significant outbreaks in 2010 caused national concern. Since 2011, HFMD has been included in the National Communicable Disease Surveillance System. According to data from the Viet Nam Ministry of Health in 2012, HFMD had the highest mortality among the notifiable communicable diseases under the General Department of Preventive Medicine, Ministry of Health, with 157 391 cases and 45 deaths. (*5*)

Certain meteorological factors have been found to be associated with the occurrence of HFMD. Temperature had a positive association with the number of HFMD cases in studies. (*6*-*9*) In Japan, the weekly number of HFMD cases rose by 11.2% when average temperatures increased 1 °C. (*10*) The relationship with humidity was inconsistent; some studies showed the risk of HFMD increased 0.51–4.7% when relative humidity elevated 1%, (*6*, *8*-*10*) while other studies reported that HFMD and humidity were not associated. (*11*, *12*) The relationship between HFMD and rainfall is also inconsistent. A study in Guangdong supported a positive association between rainfall and HFMD, (*6*) while two studies from Guangdong found a non-significant association. (*8*, *9*) In China, when wind speed increased 1 m/s, the risk of HFMD increased 4.01%. (*9*) A study in Hong Kong Special Administrative Region SAR (China) also demonstrated that wind speed was positively associated with HFMD consultation rates. (*11*) Most studies denoted positive associations with evaporation and sunshine and HFMD occurrence. (*6*, *11*, *13*)

In Viet Nam, the association between HFMD and climate parameters has not been well examined. A model including climate parameters could be used as an early surveillance system to predict annual HFMD epidemics. (*14*) This study aimed to describe the occurrence of HFMD, and its association with meteorological factors in Dak Lak province in the Central Highlands region of Viet Nam.

## Methods

### Study setting

**Figure 1:**
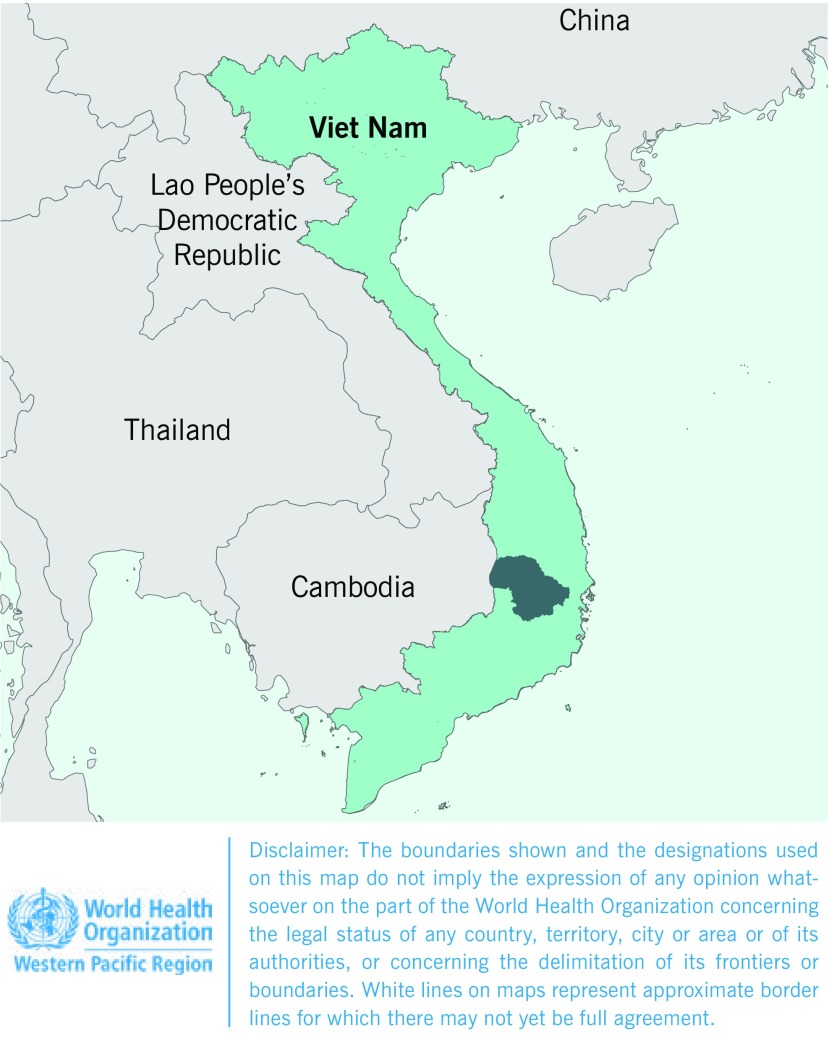
**Map of Viet Nam showing Dak Lak province (in dark green area)**

An ecological study was conducted using data from January 2012 through December 2013 in Dak Lak province (total population: ~1.8 million). Dak Lak is located between 12°09′–13°25′ north latitudes and 107°28′–108°59′ east longitudes and shares a border with Cambodia (**Fig. 1**). The terrain is mainly relatively flat highland with an average altitude of about 500 m above sea level. Dak Lak has a tropical monsoon climate with two distinct seasons: the rainy season is usually from May through October and the dry season is from November through April. The rainy season typically receives 90% of the annual rainfall. The annual average rainfall is about 2000 mm, and the annual average temperature ranges between 23 °C and 24 °C. (**Table 1**)

[**insert** Figure 1]

**Table 1:** Climate change and occurrence of hand, foot and mouth disease cases stratified by month, Dak Lak  province, Viet Nam, 2012–2013

Month	No. of cases (a)	Average temperature (°C) (b)	Maximum temperature (°C) (b)	Minimum temperature (°C) (b)	Humidity (%) (b)	Rainfall (mm) (b)	Evaporation (mm) (b)	Sunshine (hours) (b)	Wind speed (m/s) (b)
January	261	20.8	29.9	15.2	82.0	15.4	96.1	198.1	2.9
February	239	22.5	33.1	16.5	79.0	9.4	105.0	224.5	2.7
March	436	24.3	34.0	16.8	76.7	50.9	124.4	248.4	2.4
April	604	25.5	34.6	20.0	79.1	170.5	101.1	233.4	1.7
May	658	25.6	33.5	20.3	81.5	193.7	89.5	252.9	1.4
June	504	24.7	31.4	20.7	85.7	223.4	68.1	163.1	2.1
July	427	24.2	31.5	19.7	86.8	206.2	65.5	163.8	1.8
August	527	24.2	31.5	20.2	85.3	184.8	73.5	166.3	2.4
September	1017	23.7	31.1	20.0	88.0	525.3	52.0	131.9	1.8
October	1285	23.2	30.2	18.0	84.8	163.1	69.4	182.9	1.8
November	849	23.2	30.6	17.6	85.3	97.5	72.8	188.9	2.3
December	321	20.9	29.2	14.9	82.7	16.6	87.0	202.1	2.5

a Data are total number of cases tallied from 2012 through 2013.

b Data are averages across two years (2012–2013).

HFMD prevention and control activities in Dak Lak province were carried out under an unofficial multisectoral committee. Key activities included surveillance of HFMD with routine weekly reports, laboratory-based sentinel surveillance and monitoring of environmental risk factors for HFMD epidemics.

### Data collection

The number of HFMD cases was collected from the Center for Disease Control of Dak Lak Province. These data were obtained through the Communicable Disease Surveillance System in Viet Nam from 2012 through 2013. (*15*) Circular 54/2015/TT-BYT mandates the reporting of HFMD by all levels of health care, from the commune health station to the national level.

According to the Viet Nam Ministry of Health protocol, (*15*) mainly based on WHO recommendations, (*1*) individuals suspected of having HFMD were those who meet the case definition as a brief febrile illness accompanied by a typical skin rash with or without mouth ulcers. Once identified, a patient was treated at the nearest health facility or transferred, depending on the severity of the condition, to a district or provincial hospital for further diagnosis and treatment. Total numbers of HFMD cases were recorded monthly during the surveillance period from 2012 through 2013. Meteorological data were provided by the hydro-meteorological forecast station of Dak Lak province. (*16*) Average/maximum/minimum temperature (°C), relative humidity (%), amount of rainfall (mm), amount of evaporation (mm), duration of sunshine (hours) and average wind speed (m/s) were recorded daily from five stations of meteorology throughout Dak Lak province and averaged for each month.

### Data analysis

The main aim of the data analysis was to determine if an association exists between the number of HFMD cases and the meteorological parameters. The outcome was the monthly number of HFMD cases in each district. The predictive variables were average temperature, maximum temperature, minimum temperature, humidity, rainfall, evaporation, sunshine duration and wind speed.

The study assumed that the distribution of HFMD cases followed the Poisson distribution as the number of HFMD cases was relatively small compared to the provincial population. Poisson regression was used to model the associations between the meteorological factors and the distribution of HFMD cases. Due to a variation of meteorological factors in season and location, in subsequent analyses, time (month, year) and area (district) were considered simultaneously in a multivariable model. The effects of meteorological variables were modelled as follows:

where ***β***_0_, ***β***_1_, ***β***_2_, *…,* ***β****_p_*, are regression coefficients related to variables ***x****_t_*_0_, ***x****_t_*_1_, ***x****_t_*_2_, *…,* ***x****_tp_*, respectively (with ***x****_t_*_0_ = **0)**, and **λ***_t_* denoted the number of HFMD cases at month *t*. The regression coefficients were estimated by the method of maximum likelihood by using the R program package. (*17*)

### Ethics statement

The study was approved by the Scientific Committee of the University of Medicine and Pharmacy at Ho Chi Minh City, Viet Nam as Decision No. 66/YTCC-DT dated 25 March 2014.

## Results

In 2012 through 2013, the National Disease Surveillance System reported there were 7128 HFMD patients in Dak Lak: 5191 patients in 2012 (incidence rate: 289 per 100 000 population) and 1937 patients in 2013 (186 per 100 000 population).

**Figure 2:**
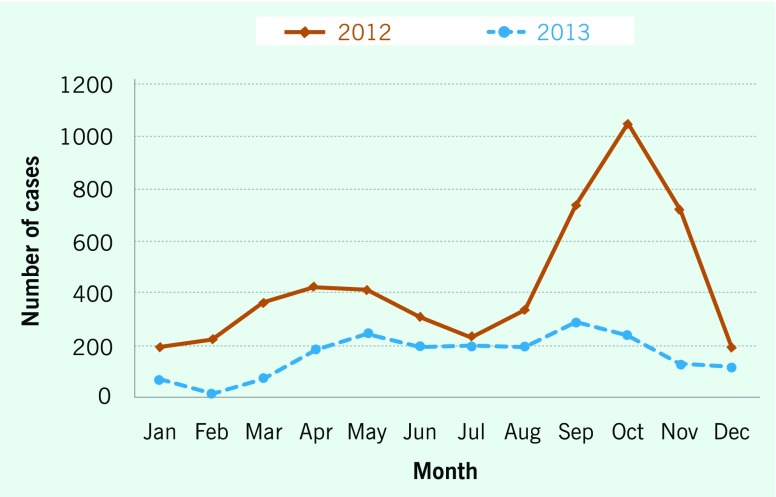
**Distribution of hand, foot and mouth disease cases by month, Dak Lak province, Viet Nam, 2012–2013**

Although HFMD patients were reported throughout the year, the number of HFMD cases increased from April through May and September through October (**Fig. 2**), accounting for about 50% of total HFMD cases. The average number of patients per month was 25 in the rainy season (from May through October) and 15 in the dry season (from November through April of the next year). Compared to the dry season, on average, there were 10 more patients per month in the rainy season (95% CI: 4–15) cases (*P* < 0.005).

[**insert** Figure 2]

Data analysis showed that the number of HFMD cases was associated with climate factors (**Tables 2**** and ****3**). Due to multicollinearity among average temperature, maximum temperature and minimum temperature of these variables, only average temperature and humidity were included in the final model. The correlation coefficients (r) of average temperature with maximum and minimum temperature were 0.77 and 0.82, respectively; between humidity and evaporation, the correlation coefficient (r) of humidity and evaporation was 0.87.

**Table 2:** Risk factors for hand, foot and mouth disease in Dak Lak province: univariate analysis

Risk factor	Unit of comparison	Risk ratio (95%CI)	p-value
Average temperature	Per 1 °C increase	1.18 (1.16 – 1.21)	< 0.001
Humidity	Per 5% increase	1.14 (1.11 – 1.18)	< 0.001
Rainfall	Per 200 mm increase	1.26 (1.23 – 1.29)	< 0.001
Sunshine	Per 60 hours increase	0.95 (0.91 – 0.98)	< 0.001
Wind speed	Per 1 m/s increase	0.66 (0.63 – 0.69)	< 0.001

**Table 3:** Risk factors for hand, foot and mouth disease in Dak Lak province: multivariable analysis

Risk factor	Unit of comparison	Risk ratio (95%CI)	p-value
Average temperature	Per 1 °C increase	1.06 (1.03 – 1.08)	< 0.001
Humidity	Per 1% increase	1.06 (0.99 – 1.12)	0.053
Rainfall	Per 200 mm increase	1.19 (1.14 – 1.24)	< 0.001
Sunshine	Per 60 hours increase	1.14 (1.07 – 1.22)	< 0.001
Wind speed	Per 1 m/s increase	0.77 (0.73 – 0.81)	< 0.001

Results of univariate analysis showed a significant increase in the risk of HFMD when average temperature, humidity and rainfall were elevated. The study found a reverse association between HFMD and sunshine duration and wind speed. In the multivariable Poisson regression model, results indicated that average temperature, rainfall and sunshine duration had significantly positive effects on the number of HFMD cases. The risk of HFMD, however, reduced when wind speed increased. Humidity was not significantly associated with HFMD case counts in the multivariable model.

## Discussion

The present study demonstrated a seasonal pattern of HFMD occurrence in a Central Highlands province of with a higher number of cases occurring in the rainy season. This was one of a few studies examining the association between meteorological factors and HFMD occurrence in Viet Nam. Our finding is consistent with what has been reported in a previous study conducted in southern Viet Nam. (*4*) However, exact reasons for the relationship between weather and HFMD are limited. Meteorological factors could affect occurrences of infectious disease via survival and transmission of pathogens in the environment as well as population activities and behaviour. (*18*-*20*)

Pathophysiology of enteroviruses was found to be affected by temperature, humidity and surface of fomites. (*19*) This study found that within the range of average temperatures in the region, a one-degree higher average temperature was associated with an increase of 6% in the number of HFMD cases. Studies from Hong Kong Special Administrative Region SAR (China) and Japan revealed similar findings: a positive association between average temperature and number of HFMD cases. (*7*-*11*, *20*-*22*) Moreover, a study in Hong Kong Special Administrative Region SAR (China) showed that warm weather in winter might increase the number of HFMD cases. (*23*) High temperatures could increase the growth of enteroviruses and also interfere with inactivation and recovery of enteroviruses. (*24*, *25*)

HFMD has seasonality. In temperate regions, the number of patients who are infected with enteroviruses rises in summer. (*11*, *25*, *26*) In subtropical and tropical regions, enteroviruses circulate throughout the year and elevate during the rainy season. (*1*) This study found each 200 mm increase in rainfall was associated with a 19% increased risk of HFMD onset. This finding is also consistent with that found in previous studies from other countries. (*6*, *27*, *28*) A possible explanation is that high rainfall makes soil moist, which may facilitate viral persistence and spreading. (*25*, *29*) In contrast, some studies in China did not support the association between rainfall and HFMD. (*8*, *9*)

Although some previous studies suggested that humidity was associated with HFMD, (*6*-*8*, *10*, *11*) the current study did not find a statistically significant association between humidity and the number of HFMD cases each month. The difference between the current study and previous studies that showed a positive effect was the use of monthly data. Another study using monthly data also concluded no association with the number of HFMD cases. (*27*)

In agreement with results from other studies that showed the effect of increased sunshine, (*12*, *30*) our findings showed that the risk of HFMD increased by 14% per 60 hours of increase in sunshine duration. However, another study showed a negative correlation between sunshine duration and HFMD infection; (*2*) this disparity needs further researches to provide more evidence. For wind speed, this study denoted a negative association with the number of HFMD cases: 1 m/s increase in wind speed leads to a decrease of 23% in the risk of HFMD. A possible reason is that months with higher wind speed in Dak Lak were often from December through February, which is the dry season with lower temperature. These factors could have an effect on the dispersal and persistence of pathogens in the environment.

The current study had some limitations: HFMD epidemics have been shown to occur in two- to three-year cycles, (*30*) and the two-year period in our study might not be adequate to identify the cycle of enteroviruses and the effects of climate change on HFMD in an ecological analysis. It would be useful to conduct a longer study and conduct time series analysis to detect the natural cycle of HFMD outbreaks in this region. Databased on surveillance systems might be underestimated. To our knowledge, there were several HFMD patients treated in private clinics that were not recorded. In addition, HFMD patients with mild self-limiting or unclear symptoms were not diagnosed as HFMD and were not notified to the HFMD surveillance system.

## Conclusion

HFMD is a seasonal health-related challenge in Dak Lak province and other geographical areas with the same climatic characteristics. Understanding the association between HFMD and meteorology is important to predict epidemic trends. Future studies should explore the association between other meteorological factors and the incidence of HFMD to provide more evidence for new policies to be developed. Health departments should use more meteorological data to predict the number of HFMD cases, to identify periods of high risk for HFMD outbreaks and increase health communications during outbreaks. The data also suggest that the occurrence of HFMD in this region is likely the result of multiple causes that remain to be delineated; we recommend that research be conducted to describe a more complete picture of risk factors for HFMD development.
